# Hepatic dysfunction among scrub typhus Indian patients with acute undifferentiated febrile illness

**DOI:** 10.6026/973206300210137

**Published:** 2025-02-28

**Authors:** Sourabh Dwivedi, Kapila Gaikwad, Yar Mohammad Ansari, Pratiksha Pandey Dwivedi, Shikhar Sharma

**Affiliations:** 1 Department of Biochemistry, Shyam Shah Medical College, Rewa, Madhya Pradesh, India; 2 Department of Microbiology, Rajmata Shrimati Devendra Kumari Singhdeo Government Medical College, Ambikapur, Chhattisgarh, India; 3 Department of Community Medicine, Shyam Shah Medical College, Rewa, Madhya Pradesh, India

**Keywords:** Scrub typhus, hepatic dysfunction, acute liver failure, age group, days of fever

## Abstract

Scrub typhus is a febrile illness caused by through trombiculid mite, bite *Orientia tsutsugamushi*. It is often unrecognized due to its vague symptoms. It is prevalent in the Rewa region of Madhya Pradesh, India. Therefore, it is of interest to assess hepatic dysfunction and to establish the association between liver function test parameters and disease severity in confirmed Scrub typhus cases. Hence, 50 IgM ELISA positive cases from July to September 2024 at Shyam Shah Medical College, Rewa were studied. Data were analysed using IBM SPSS 25 software, with Spearman's correlation used to relate severity, liver function test parameters and fever duration. The average fever duration was 6.22 days, with 80% of patients showing serum glutamate oxaloacetate transaminase and 70% showing serum glutamate pyruvate transaminase abnormalities. Thus, there was a correlation between disease severity and liver dysfunction, with moderate injury in 62% and severe injury in 38% of patients.

## Background:

The bacteria *Orientia tsutsugamushi* is the cause of scrub typhus, a potentially fatal disease. In northern India, it is responsible for 11.2-14.4% of acute febrile diseases [1]. India is facing a health crisis, with high disease cases in Tamil Nadu andhra Pradesh, Karnataka, Kerala, Himachal Pradesh, Uttarakhand, Jammu, Kashmir, Meghalaya, Assam, Nagaland, West Bengal, Bihar, Maharashtra and Rajasthan [8, 9]. In rural India and Southeast Asia, scrub typhus cases are often reported in large numbers during the monsoon and post-monsoon seasons (June to November) [9, 10]. The bacteria *Orientia tsutsugamushi* is the cause of the zoonotic disease scrub typhus. The primary mode of transmission occurs through the bite of the larval stage of mites, particularly those belonging to the Leptotrombidium species. Rodents often serve as common animal hosts for scrub typhus. Humans typically contract the disease after being bitten by the larval stage of these mites, known as chiggers, particularly during recreational or agricultural activities [4]. Scrub typhus is definitively caused by the bacterium Orientia tsutsugamushi, which is found in Leptotrombidium mites [2, 3]. It causes a wide range of intricate clinical presentations, frequently coupled with different comorbidities [4, 5 and 6]. Patients often seek medical attention after a median of eight days [1, 7], presenting with symptoms that range from uncomplicated febrile illness to severe mono - or multi-organ dysfunction, which can tragically lead to fatal outcomes. In the absence of proper diagnosis and treatment the patient often lands up in critical MODS (multi organ dysfunction syndrome) [1]. The following signs and symptoms may be observed Fever and chills, Headache, Body aches and muscle pain, a dark scab like area at the site of a chigger bite (referred to as eschar), Mental changes, which can range from confusion to coma, Enlarged lymph nodes, Rash [12].

It is important to identify predictors that can mark severe cases of disease to reduce mortality, particularly due to the high incidence of severe manifestations and complications, as well as delays in treatment. By recognizing these markers, patients at risk of severe disease can be admitted to hospitals or transferred to well-equipped facilities earlier. Close monitoring and intensive care can prevent complications and reduce severe morbidity or mortality. Marker linked to severe scrub typhus is known [11]. Therefore, it is of interest to assess hepatic dysfunction and to establish the association between liver function test parameters and disease severity in confirmed Scrub typhus cases.

## Materials and Methods:

It is a comprehensive prospective observational study conducted at tertiary care centre Shyam Shah Medical College, Rewa, Madhya Pradesh over three-month period from July 2024 to September 2024 and ethical approval was taken.

## Inclusion criteria:

The participants in this study encompass a diverse range of age groups, including children, adults and the elderly, who initially presented with symptoms of acute undifferentiated febrile illness (AUFI) like fever greater than 5 days and less than 14 days duration, headache, myalgia, arthralgia, lymphadenopathy, hepatomegaly, splenomegaly, skin rash, with or without Escher, *etc.* to the Outpatient department (OPD) and who were admitted to Inpatient department (IPD) across various medical specialties of Shyam Shah Medical College in Rewa, Madhya Pradesh. Suspected cases of scrub typhus were subjected to serological testing to confirm the presence of anti - *Orientia tsutsugamushi* antibodies at microbiology laboratory and information regarding IgM ELISA positive cases (using J Mitra kit) was retrieved from there only. Patients subsequently underwent a detailed biochemical analysis to assess liver function and those who had given written informed consent are included in this study.

## Exclusion criteria:

Patient who was tested negative for Scrub typhus and those who were tested positive for other causes of acute undifferentiated febrile illness like Dengue, Chikungunya, Leptospirosis, Urinary tract infection, tuberculosis *etc.* and those who do not gave consent for the study were excluded.

## Sample collection and testing:

Data collection was meticulously carried out in the laboratories, including the VDRL section of the Department of Microbiology and the central laboratory of the Department of Biochemistry at SSMC. Whole blood sample was collected from scrub typhus positive cases in red cap vacationer and separated serum sample were used to analyse the parameters included in this study such as serum glutamate oxaloacetate transaminase (SGOT or AST), serum glutamate pyruvate transaminase (SGPT or ALT), serum alkaline phosphatase levels, total Serum Bilirubin and Serum Albumin in Beckman dxc 700 au biochemistry analyser. These liver function test parameters were critical for evaluating the extent and severity of liver dysfunction in patients diagnosed with scrub typhus.

## Statistical analysis:

To effectively analyse the data collected, we employed IBM SPSS version 25, widely recognized and robust statistical analysis software. In addition to basic descriptive statistics, we established correlations between the severity grading of the disease and various liver function test parameters. We also examined the relationship between these laboratory values and the duration of fever experienced by the patients. For this purpose, we utilized Spearman's correlation coefficient, a non-parametric measure that allowed us to assess the strength and direction of the associations between multiple variables while accounting for any potential non-linear relationships.

## Results:

We included 50 patients, ages ranging from infancy to 80 years, who had positive scrub typhus tests in our study. In order to comprehend the impact of sickness severity on liver function. Fever lasted 6.22 days on average for the patients and this was the case in 30% of instances.

Several noteworthy facts were shown by the liver function test results are Serum glutamate-oxaloacetate transaminase (SGOT) was abnormal in 80% of cases. Serum glutamate-pyruvate transaminase (SGPT) was abnormal in 70% of cases. Serum alkaline phosphatase (ALP) was abnormal in 50% of cases. Serum bilirubin levels were high in 62% of the cases. Serum albumin levels were abnormal in 54% of patients. These results point to a major impact on liver function in these patients. We analyzed the relationship between severity and liver function test results. Table 1 shows serum glutamate oxaloacetate transaminase had a correlation coefficient of 0.288, with a significance level of p = 0.042. Serum glutamate pyruvate transaminase had a stronger correlation at 0.326, with p = 0.021. ALP had a correlation of 0.315, with p = 0.026. Total bilirubin had a correlation of 0.448 and a significant p-value of 0.001. These results suggest that as disease severity increases, levels of liver enzymes and bilirubin also rises, indicating worsening liver function. We also found strong relationships among liver enzymes.

Serum glutamate oxaloacetate transaminase and serum glutamate pyruvate transaminase had a strong correlation of 0.803 (p < 0.001), indicating that when serum glutamate oxaloacetate transaminase levels rise, serum glutamate pyruvate transaminase levels do too. Serum glutamate oxaloacetate transaminase also showed a moderate correlation with serum total bilirubin at 0.336 (p = 0.017), meaning higher serum glutamate oxaloacetate transaminase levels are linked to increased bilirubin, a sign of liver dysfunction. Serum glutamate pyruvate transaminase had a positive correlation with ALP at 0.313 (p = 0.027), showing that as serum glutamate pyruvate transaminase rises, alkaline phosphatase levels increase as well. However, serum albumin did not show significant correlations with the other parameters, indicating that while liver enzyme levels and bilirubin signal worsening conditions, albumin levels remain stable (Figure 4 - see PDF).

We found at how the number of fever days relates to liver function tests. Our findings showed a significant positive relationship between the duration of fever and liver enzyme levels (Table 2). Serum glutamate oxaloacetate transaminase had a correlation of 0.349 (p = 0.013), (Figure 2 - see PDf) indicating that longer fever duration relates to higher serum glutamate oxaloacetate transaminase levels. serum glutamate pyruvate transaminase had a correlate of 0.326 (p = 0.021), further suggesting that longer fever durations are linked to higher serum glutamate pyruvate transaminase levels (Figure 1 - see PDF). Serum glutamate oxaloacetate transaminase and serum glutamate pyruvate transaminase showed a very strong correlation (0.803, p < 0.001), meaning that both enzymes rise together during fever. Serum glutamate oxaloacetate transaminase also had a moderate correlation with total bilirubin (0.336, p = 0.017), (Figure 5 - see PDF) suggesting a link between higher serum glutamate oxaloacetate transaminase levels and increased bilirubin. Finally, serum glutamate pyruvate transaminase positively correlated with serum alkaline phosphatase (ALP) (Figure 3 - see PDF). Our results showed a strong link between the severity of scrub typhus and various liver function tests (LFT) results. As the illness gets worse, liver function also declines. This highlights the need to monitor liver function in scrub typhus patients for better care and treatment.

## Discussion:

The rising incidence of scrub typhus in India has become a pressing public health issue. This increase may be attributed to factors such as previous under-reporting and misdiagnosis, which often arise from a general lack of awareness among healthcare professionals about this serious condition. Moreover, the rapid urbanization of rural areas, combined with enhanced transportation options due to economic development, calls for thorough examination, as these elements may facilitate the disease's spread. Healthcare providers must remain vigilant; any patient presenting with fever along with liver or kidney dysfunction should be thoroughly evaluated for scrub typhus, particularly during the post-monsoon season when the risk is notably higher. The economic impact of this disease is significant, especially since it tends to affect the working-age population, many of whom are regularly engaged in outdoor activities. This not only poses health challenges for individuals but also places a considerable burden on economic productivity and healthcare resources across the nation. Historically, scrub typhus was thought to primarily occur in rural regions, where conditions such as agriculture and natural habitats were conducive to the presence of the disease's vector, the mite [13]. However, recent observations indicate that scrub typhus has become endemic throughout India, affecting a much broader geographic range. Outbreaks are now being reported not only in traditional rural areas but also in metropolitan cities, where urbanization and environmental changes may be contributing to the spread of this infectious disease. This shift highlights the growing need for heightened awareness and public health measures to address scrub typhus in both urban and rural settings [14, 15]. The severity of illness associated with scrub typhus is known, to enhance our understanding of how scrub typhus affects individuals and the underlying predictors of hepatic complication that contribute to its more severe manifestations. The majority of research studies are conducted in the medicine ward and its corresponding intensive care unit (ICU). These investigations focus on patients presenting with positive IgM ELISA results in their scrub reports, specifically those who are experiencing hepatic complications. The patients presented to the hospital after experiencing average illness duration of 5 days. During this period, they reported a spectrum of troubling symptoms. These included a persistent high fever, which often fluctuated, along with nausea that led to frequent episodes of vomiting. Many patients described a feeling of shortness of breath, which became pronounced with minimal exertion. Additionally, they exhibited a troubling cough that varied in severity throughout the day. Complaints of intense headaches were common; this combination of symptoms prompted their urgent medical evaluation. The pathogenesis begins with the bacterium spreading throughout the body from the initial site of bite. This systemic dissemination leads to the infection of endothelial cells, which line the blood vessels. As these cells become infected, they sustain damage, resulting in vasculitis-an inflammation of the blood vessels. This inflammatory response can disrupt normal blood flow and compromise the functioning of various organs, ultimately leading to significant organ dysfunction [15, 16]. The symptoms of an infection are influenced by both the specific strain of the pathogen and the host's immune response. Clinically, this condition can closely resemble other tropical illnesses like dengue, malaria, acute viral hepatitis and leptospirosis. It often presents as a febrile illness and carries the risk of severe complications, including acute kidney injury, hepatitis, meningo-encephalitis, disseminated intravascular coagulation, myocarditis and shock. Early recognition and intervention are crucial for effective management and better patient outcomes.

We have identified hepatic manifestations in a patient with no prior history of liver dysfunction, highlighting a crucial link between the duration of fever and liver impairment in scrub typhus cases. Additionally, we evaluated the severity of hepatic dysfunction, revealing notable changes in SGOT, serum glutamate pyruvate transaminase and Serum bilirubin levels. While Serum alkaline phosphatase (ALP) showed only minimal alterations and Serum albumin levels remained stable, these findings underscore the need for careful monitoring of liver function in such patients, emphasizing the importance of timely diagnosis and management. Rajapakse *et al.* found significant health concerns, with 88.6% (156 out of 176) of participants showing elevated AST levels (normal < 40 IU/ml) and 90.3% (159 out of 176) with elevated ALT levels (normal < 40 IU/ml). These results highlight the urgent need for monitoring liver health. Clinically detectable hepatomegaly was present in 22% (39/176) [15]. Sivarajan *et al.* significant abnormalities were identified in 11 out of 90 patients presenting with jaundice. These patients predominantly exhibited conjugated hyperbilirubinemia, coupled with elevated liver enzyme levels that warrant attention. Notably, the median AST level reached 460 IU/L (IQR: 207-627), clearly indicating that AST levels were consistently higher than ALT, which had a median of only 102 IU/L (IQR: 86-109.5). This finding, with an AST/ALT ratio exceeding 1, underscores the potential severity of liver dysfunction. Furthermore, alkaline phosphatase (ALP) levels were markedly elevated in most patients, highlighting the need for thorough evaluation and management in such cases [17]. Takhar *et al.* in Rajasthan found a mortality rate of 21.2%, with elevated transaminases in 48.5% of cases, underscoring the urgent need for targeted medical interventions [18]. Kundu *et al.* raised aspartate transaminase (AST) and alanine transaminase (ALT) were observed in 71.1% and 64.4% of cases, respectively, while hypoalbuminemia was noted in 78.9% of participants [19]. Samanta *et al.* by 150 patients of paediatric age group multivariate analysis identified thrombocytopenia as an independent positive predictive factor for acute hepatitis caused by tropical infections, with an odds ratio (OR) of 4.237. In contrast, significantly elevated levels of total bilirubin (OR 0.575), direct bilirubin (OR 0.498), aspartate aminotransferase (OR 0.841) and alanine aminotransferase (OR 0.863) were found to be independent negative predictive factors for the same condition [20] Sivarajan *et al.* In a study of 90 jaundiced patients, 11 had conjugated bilirubinaemia and elevated liver enzymes, with a median AST of 460 IU/L and ALT at 102 IU/L. Most also had high alkaline phosphatase (ALP) levels [21]. Kumar *et al.* the clinical profile of pregnant patients with scrub typhus is effectively similar to that of non-pregnant patients [22].

Yadav *et al.* patients had elevated liver enzymes: aspartate aminotransferase (88.9%), alanine aminotransferase (70.4%) and alkaline phosphatase (85.2%). Hyperbilirubinemia occurred in 13 patients (48.1%) [23]. Kumar *et al.* (62.9%) patients had clinical jaundice in a study conducted in Madhya Pradesh India [24]. Yang *et al.* concludes that hepatocellular damage is absorbed in cases of scrub typhus which is in conjunction with the results of our study [25]. In our research, we discovered that liver dysfunction can be categorized into various levels of severity, ranging from non-severe to very severe. This categorization is based on the elevation of specific enzyme levels associated with liver function, which aligns with findings from other studies in the field. We are aware that our study has some limitations, but these do not overshadow its valuable contributions. It was conducted over a relatively short period and at a single medical centre, which may impact the applicability of our results. As a result, caution should be exercised when attempting to generalize these findings to a broader patient population.

## Conclusion:

Many scrub typhus patients show signs of liver dysfunction with elevated liver enzymes. There is an urgent need for awareness and timely intervention where 62% experiencing moderate and 38% severe acute liver injury. Thus, we can improve diagnosis and patient management leading to better outcomes for affected individuals by understanding these clinical manifestations better.

## Figures and Tables

**Table 1:** Comparison of hepatic biochemical parameters and outcomes of scrub typhus with grading of severity of liver injury

		**Spearman's correlation between days of fever & LFT parameters**					
		**Days of fever**	**SGOT**	**SGPT**	**Serum ALP**	**Serum Albumin**	**Serum Total Bilirubin**
Days of fever	Correlation Coefficient (ρ)	1	.349*	.326*	0.073	-0.248	0.02
	p value		0.013	0.021	0.615	0.083	0.89
SGOT	Correlation Coefficient (ρ)	.349*	1	.803**	0.246	-0.036	.336*
	p value	0.013		0	0.085	0.803	0.017
SGPT	Correlation Coefficient (ρ)	.326*	.803**	1	.313*	-0.045	0.223
	p value	0.021	0		0.027	0.754	0.12
Serum ALP	Correlation Coefficient (ρ)	0.073	0.246	.313*	1	-0.132	0.001
	p value	0.615	0.085	0.027		0.363	0.995
Serum Albumin	Correlation Coefficient (ρ)	-0.248	-0.036	-0.045	-0.132	1	0.069
	p value	0.083	0.803	0.754	0.363		0.636
Serum Total Bilirubin	Correlation Coefficient (ρ)	0.02	.336*	0.223	0.001	0.069	1
	p value	0.89	0.017	0.12	0.995	0.636	
	N	50	50	50	50	50	50
*. Correlation is significant at the 0.05 level (2-tailed).							

**Table 2:** Comparison of hepatic biochemical parameters and outcomes of scrub typhus with days of fever

		**Spearman's correlation between severity grading & liver function test parameters**					
		**Severity Grading**	**SGOT**	**SGPT**	**Serum ALP**	**Serum Albumin**	**Serum Total Bilirubin**
Severity Grading	Correlation Coefficient (ρ)	1	.288*	.326*	.315*	0.165	.448**
	p value		0.042	0.021	0.026	0.252	0.001
SGOT	Correlation Coefficient (ρ)	.288*	1	.803**	0.246	-0.036	.336*
	p value	0.042		0	0.085	0.803	0.017
SGPT	Correlation Coefficient (ρ)	.326*	.803**	1	.313*	-0.045	0.223
	p value	0.021	0		0.027	0.754	0.12
Serum ALP	Correlation Coefficient (ρ)	.315*	0.246	.313*	1	-0.132	0.001
	p value	0.026	0.085	0.027		0.363	0.995
Serum Albumin	Correlation Coefficient (ρ)	0.165	-0.036	-0.045	-0.132	1	0.069
	p value	0.252	0.803	0.754	0.363		0.636
Serum Total Bilirubin	Correlation Coefficient (ρ)	.448**	.336*	0.223	0.001	0.069	1
	p value	0.001	0.017	0.12	0.995	0.636	
	N	50	50	50	50	50	50
*. Correlation is significant at the 0.05 level (2-tailed).							
